# Case report: Curative effect and mechanism of surufatinib combined with toripalimab in the treatment of 1 case of lung large-cell neuroendocrine carcinoma

**DOI:** 10.3389/fonc.2025.1527719

**Published:** 2025-09-03

**Authors:** Shuanglong Xiong, Chengxiang Yang, Kaiwen Fu, Xingyun Liao, Yao Ding, Kexue Zhou, Li Zhang, Yan Teng, Chunman Wu, Yongsheng Li, Houjie Liang, Yan Li

**Affiliations:** 1 Department of Oncology, Chongqing University Cancer Hospital, Chongqing, China; 2 Nanjing Geneseeq Technology Inc, Nanjing, China; 3 Department of Oncology and Southwest Cancer Centre, Southwest Hospital, Army Medical University (Third Military Medical University), Chongqing, China

**Keywords:** lung large-cell neuroendocrine carcinoma, Surufatinib, toripalimab, immune microenvironment, TMB

## Abstract

**Background:**

Large-cell neuroendocrine lung carcinoma (LCNEC) is a rare heterogeneous tumour with rates ranging from 2.1% to 3.5%. The overall prognosis of pulmonary LCNEC is poor, and the 5-year overall survival rate is only 21%. Advanced LCNEC treatment drugs are limited, and their efficacy is low. Here, we report a case of advanced lung LCNEC treated with front-line chemotherapy, which significantly delayed tumour progression after combined therapy with targeted immunotherapy. Moreover, we explore the mechanism of the correlation between dynamic changes in the immune microenvironment and therapeutic effects.

**Case presentation:**

We describe a patient with advanced pulmonary LCNEC who achieved disease remission and progression-free survival (PFS) of up to 15.1 months after chemotherapy with first-line EP and second-line FOLFIRI. In February 2022, this patient, a 58-year-old male, was diagnosed with left lung large-cell neuroendocrine carcinoma. The initial stage was cT1N2M1aIVA (right lung); however, the disease progressed after 6 cycles of EP chemotherapy, and the revised diagnosis was left lung large-cell neuroendocrine carcinoma, stage cT4N3M1a IVA (right lung). The patient participated in the “randomized, open, multicentre phase III clinical study to evaluate the efficacy and safety of solantinib combined with toripalimab versus FOLFIRI as second-line treatment for advanced neuroendocrine carcinoma”, and the efficacy of the FOLFIRI regimen was evaluated after 4 cycles of chemotherapy for PD. The best results were a partial response (PR) and a PFS of up to 15.1 months.

**Conclusion:**

This case confirmed the efficacy of ICI therapy against TMB-H lung large-cell neuroendocrine carcinoma, suggesting that the immune microenvironment and TMB analysis are helpful for guiding the personalized treatment of LCNEC.

## 1 Introduction

Lung large-cell neuroendocrine carcinoma (LCNEC) is a rare lung malignancy that accounts for approximately 3% of all lung cancers. It is characterized by large cells with neuroendocrine characteristics and a low karyotoplasmic ratio, with obvious aggressive behaviour (1). Owing to the lack of strong clinical evidence, there is currently no standard treatment for advanced pulmonary LCNEC. In addition, owing to the rarity of this type of tumour, it is very difficult to carry out prospective studies with large samples.

At present, the main treatment for locally advanced or metastatic pulmonary LCNEC is chemotherapy. On the basis of the positive results obtained from neoadjuvant therapy and adjuvant treatment with a small-cell lung cancer (SCLC) regimen (etoposide combined with platinum), we recommend the use of an “SCLC”-based regimen in patients with unresectable and advanced lung LCNEC. However, LCNEC is more aggressive than most NSCLCs are and is less responsive to SCLC regimens (2). A multicentre retrospective study from China revealed that patients with LCNEC had PFS times of only 11.5 and 7.2 months when receiving “SCLC”-based regimens and “NSCLC”-based regimens, respectively (3).

In the past decade, targeted immunotherapy has become the primary treatment for advanced NSCLC. By blocking immune checkpoints and their ligands, immune checkpoint inhibitors (immuno-checkpoint inhibitors, ICIs) eliminate immune function, thus activating immune cells that play an antitumour role (4). However, LCNEC is a rare tumour, and clinical trials are difficult to conduct. To date, LCNEC patients have no clear indications of immunotherapy or targeted therapy. Currently, small-sample retrospective analyses and case reports have shown that LCNEC patients have higher levels of PD-L1 expression than other neuroendocrine tumours (especially SCLC) do (5), which suggests that immunotherapy may be effective for some LCNEC patients. A retrospective analysis revealed that the median overall survival (mOS) of LCNEC patients treated with ICIs was significantly longer (23.5 months vs. 11.2 months) than that of LCNEC patients who did not receive ICIs (6). A real-world study analysed the efficacy of chemotherapy combined with ICIs in advanced lung LCNEC patients, and the results revealed that the mOS times of patients in the combined treatment group and the chemotherapy group were 13.3 months and 6.6 months, respectively, and the median PFS times were 7.6 months and 4.7 months, respectively (7).

Antiangiogenic therapy can promote the normalization of abnormal blood vessels and reshape the tumour microenvironment. It can also increase the infiltration of immune effector cells into tumours and improve the effectiveness of immunotherapy. The combined application of antiangiogenic therapy and immunotherapy can have a synergistic effect, and significant benefits have been observed in the treatment of various tumour types, such as lung cancer, liver cancer, kidney cancer and breast cancer, with satisfactory overall safety (8). IM power150 (9) demonstrated that ICI combined with anti-VEGF and chemotherapy resulted in higher overall survival (OS) compared to anti-VEGF and chemotherapy alone (19.2m vs 14.7m). Moreover, regardless of PD-L1 expression levels or genetic mutation status, the combination of anti-angiogenic drugs and immunotherapy provided significant PFS and OS benefits to patients. The ETER701 Phase 3 trial (10) showed that the combination of becavirumab and anlotinib with the EC regimen significantly improved OS survival rates compared to the EC regimen alone in SCLC. These clinical studies further confirmed the synergistic effects of anti-angiogenic drugs and immune checkpoint inhibitors in promoting tumor vascular normalization and stimulating immune activation. Sovantinib, a multi-kinase inhibitor targeting VEGFR 1-3, FGFR 1, and CSF-1R, has been approved in China for the treatment of advanced or metastatic extra-pancreatic neuroendocrine tumors. Toripalimab is a monoclonal humanized IgG4 PD-1 antibody. Sovantinib modulates tumor immune microenvironment and has shown promising antitumor activity in combination with toripalimab in solid tumors, including neuroendocrine tumor and neuroendocrine carcinoma. A multicenter, open-label, single-arm, Phase II trial (11) explored the efficacy and safety of sovantinib combined with toripalimab in treating advanced neuroendocrine tumours (NETs) and neuroendocrine carcinomas (NECs) +mixed neuroendocrine non-neuroendocrine neoplasms (MiNEN) patients. The objective response rate (ORR) was 21.1% (95% CI 6.1-45.6) in the NET cohort and 23.8% (95% CI 8.2-47.2) in the NEC-MiNEN cohort. The corresponding median duration of response (DoR) was 7.1 months (95% CI 6.9-NE) and 4.1 months (95% CI 3.0-NE), respectively.

In this paper, we describe a patient with advanced pulmonary LCNEC whose disease progression was delayed by ICI therapy combined with posterior line treatment, and the PFS reached 15.1 months. This case suggests the synergistic anti-tumour effect of ICIs combined with antiangiogenic drugs in LCNEC. Moreover, we monitored the changes in the immune microenvironment before and after treatment and found that these changes were consistent with the antitumour efficacy in the patient.

## 2 Case presentation

**Figure 1:**
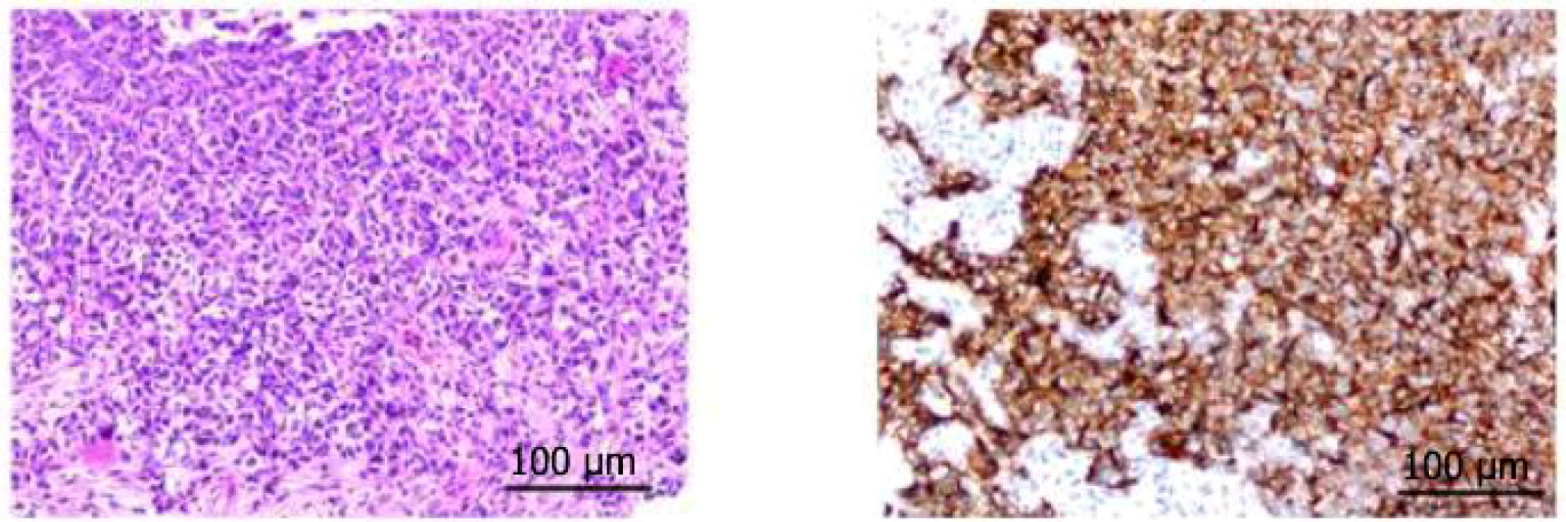
Immunohistochemical results of HE and Syn in the patient’s upper left lobe mass.

**Figure 2:**
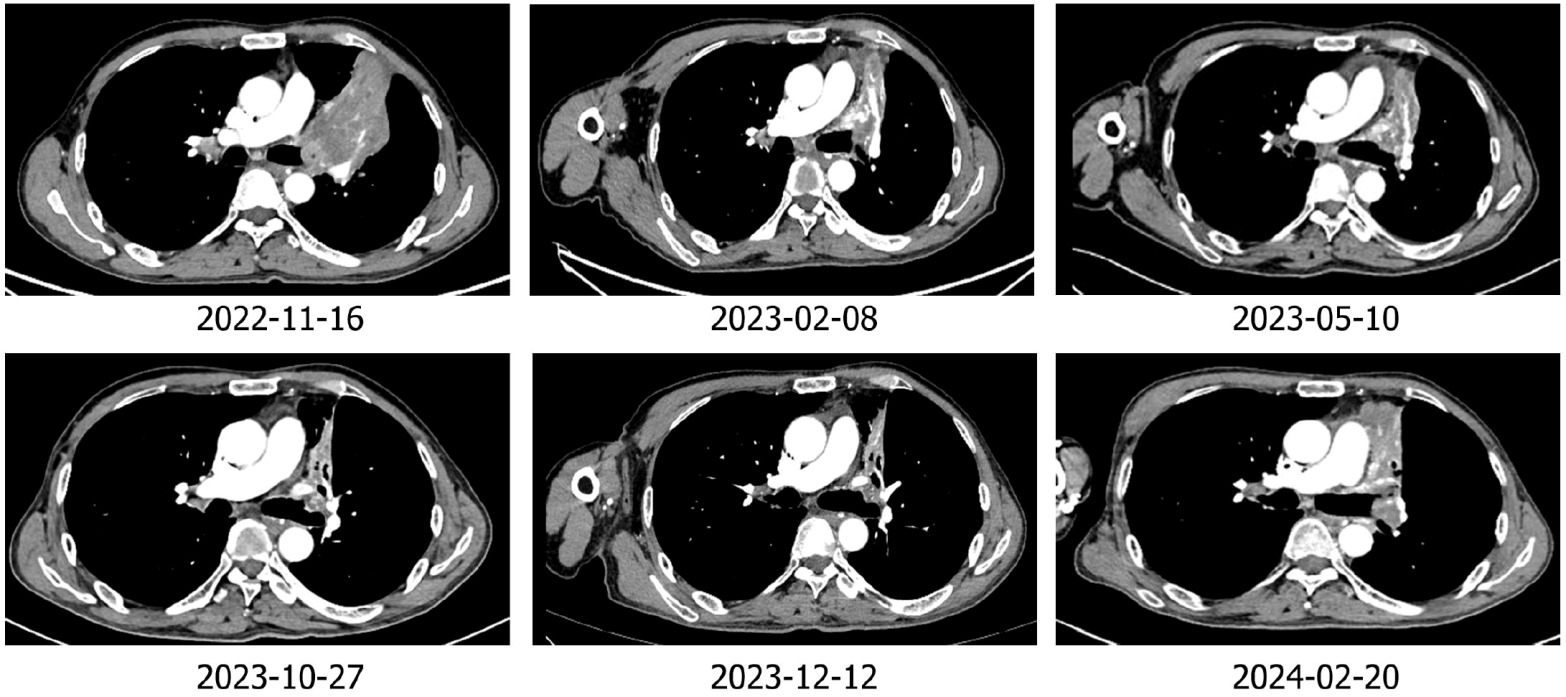
Imaging of the patient treated with toripalimab combined with surufatinib.

**Figure 3:**
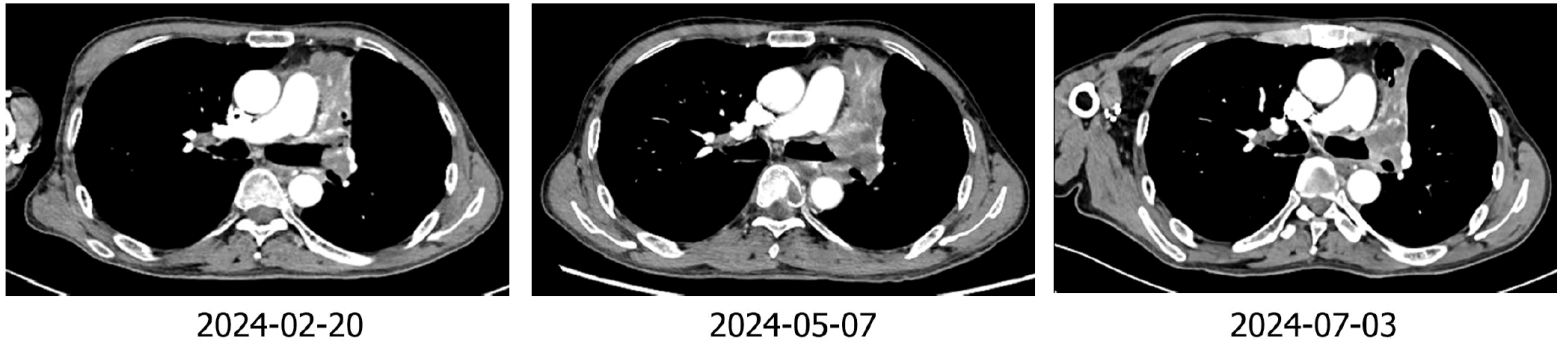
Imaging of the patient during treatment with the toripalimab-plus-EP regimen and the everolimus-plus-toripali regimen.

The patient was Li XX, 58 years old, male, smoking index 500. In 2022-02, the patient was treated for “cough”. Chest CT revealed a soft tissue shadow in the left hilum of the lung, multiple enlarged lymph nodes in the mediastinum and hilum of the lung, and nodules in the outer basal segment of the lower lobe of the right lung. Fibrobronchoscopy revealed a new protuberant lesion that was located in the opening of the upper left lobe, blocking the lumen. Forceps biopsy was performed. Pathological analysis of the specimen revealed large-cell neuroendocrine carcinoma of the upper left lobe. The immunohistochemical results were as follows: Syn (+), CD56 + (weak), CgA (-), CK (focal point +), CK5/6 (-), Ki -67 (+ 80%), NapsinA (-), P40 (-), and vera.ttf (+) - 1 (**Figure 1**). The patient was diagnosed with left lung large-cell neuroendocrine carcinoma, stage cT1N2M1aIVA (right lung). From 2022-2-18 to 2022-7-17, he received chemotherapy consisting of the EP regimen (etoposide 0.-4+ carboplatin 400 mg/d1) for 6 cycles, and the best efficacy evaluation was a PR. A review of a chest CT image taken on 2022-08-11 revealed a space-occupying lesion of the left upper hilum, which measured approximately 4.3*3.6 cm in size and was significantly larger than before. Multiple enlarged lymph nodes were observed in the mediastinum and hilum. The revised diagnosis after a complete examination was as follows: stage cT4N3M1aIVA left lung large-cell neuroendocrine carcinoma (right lung). In 2022-09, the patient was enrolled in the “randomized, open, multicentre phase III clinical study to evaluate the efficacy and safety of sofratinib combined with toripalimab versus FOLFIRI as second-line treatment for advanced neuroendocrine carcinoma”, and he was randomized to receive chemotherapy alone. Chemotherapy with the FOLFIRI regimen was performed for 4 cycles: 2022-09-09, 2022-09-24, 2022-10-09 and 2022-10-25. During chemotherapy, the patient’s gastrointestinal reaction was grade III, with no obvious bone marrow suppression. PD was evaluated after 4 cycles (target lesion: left pulmonary lesion 7.5 cm→9.3 cm), and consequently, a clinical trial was conducted. The patient was treated with sofratinib combined with toripalimab in 2022-11-18, 2022-12-30, and 2023-01-16. After 3 cycles of treatment, the mass in the upper lobe of the left lung was smaller than before (9.3 cm→7.9 cm), and the efficacy was evaluated as SD (15%↓). During the treatment period, the patient experienced no adverse events such as rash, bleeding, thrombosis, diarrhoea, abdominal pain, or constipation. Furthermore, the patient’s cough and wheezing were significantly relieved. Sofratinib combined with toripalimab was administered to the patient on 2023-02-10, 2023-03-10, 2023-04-03, and 2023-5-10 because of “aggravation of cough and sputum”, and a reexamination of the chest with enhanced CT at our hospital revealed a mass in the left upper lobe of the lung complicated with obstructive inflammation and an increased range of obstructive atelectasis (9.1 cm). Considering “lung infection”, targeted therapy was suspended. After anti-infective treatment, the patient’s cough and sputum symptoms were alleviated. A review of the patient’s chest enhanced CT imaging on 2023-5-22 revealed a mass in the upper lobe of the left lung, the overall area of which was reduced (7.9 cm), indicating effective anti-infection. On 2023-06-07, 2023-06-21, 2023-07-06, 2023-07-25, and 2023-08-14, treatment with sofratinib combined with toripalimab was continued. In 2023-08-15, CT reexamination revealed a mass in the upper lobe of the left lung with obstructive inflammation and atelectasis, the area of which was smaller than before. The efficacy was evaluated as a PR (31%↓). Treatment with sofratinib combined with toripalimab was continued, and the efficacy evaluation continued to show a PR in 2023-10-25 and 2023-12-12 (**Figure 2**). At the beginning of 2024.2, the patient felt that his cough was aggravated, and chest and abdominal CT findings were reviewed on 2024.2.20 and compared with those on 2023-12-12. CT revealed the following: 1. The mass in the upper lobe of the left lung indicated lung cancer with obstructive inflammation and obstructive atelectasis, and the overall area was larger than before (9.1×3.3 cm). 2. Compared with those in the anterior segment, the ground glass nodules in the upper segment of the right lung were newly increased, and the ground glass nodules in the posterior and anterior segments of the lower right lung were not clearly displayed this time. 3. Multiple lymph nodes in the mediastinum and both pulmonary portals showed enlargement. To indicate disease progression, the tumour was evaluated as PD (60.6%↑) (**Figure 2**). On 2024-2-22, bronchoscopy revealed a left upper lobe tumour with bleeding. Pathological results of a puncture biopsy of the left upper lobe of the lung revealed large necrosis and a small number of focal atypical cells; these findings combined with those from the clinical and immunohistochemical analyses were consistent with poorly differentiated carcinoma and high-grade neuroendocrine carcinoma, NOS. Immunohistochemical results revealed CKpan weak (+), CAM5.2 weak (+), vera.ttf (+) - 1, p40 (-), SYN (+), CGA (-), CD56 (-), P53 (+ 70%), RB1 (+), Ki - 67 (hot zone + 75%). The patient was treated with the combined EP regimen (etoposide 130 mg D1-3+ cisplatin 30 mg D1-3) on 2024-02-29 and 2024-03-22. After 2 cycles of chemotherapy, the therapeutic effect was evaluated as PD (new bone metastasis). On 2024-05-10, 2024-06-02, and 2024-06-25, the treatment regimen was replaced with everolimus combined with toripalimab for 3 cycles, while desumab was given to inhibit bone destruction. The review efficacy evaluation performed on 2024-07-03 revealed SD (21.4% ↓) (**Figure 3**); currently, the patient is continuing this new targeted immunotherapy regimen.

## 3 Preliminary study on the mechanism related to the curative effect

### 3.1 Gene mutation and TMB detection

**Figure 4:**
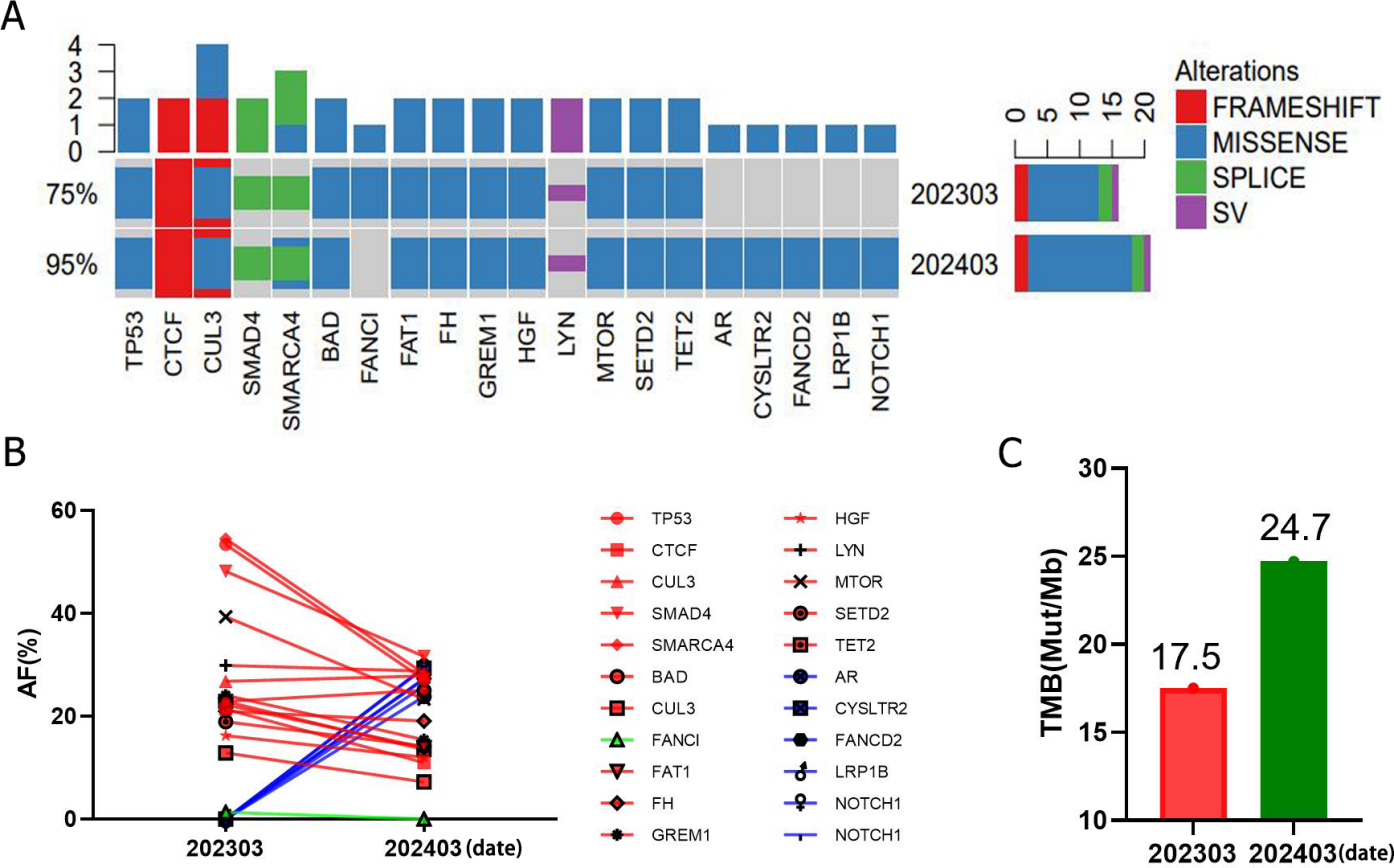
Changes in gene mutation and gene mutation abundance before and after treatment with triplelizumab combined with sofratinib [**(A)** gene mutation status during the period of treatment with sorafenib combined with toripalimab; **(B)** changes in gene mutation abundance during the combination treatment with solvatinib and toripalimumab: red indicates two common mutations, blue indicates 202,403 new mutations, and green indicates 202,303 unique mutations, and wilcoxon test was uesd to compare the gene abundance levels, and the results showed a significant difference in the median values before and after treatment with *P*=0.0006; **(C)** dynamic monitoring of TMB before and after treatment with solfaninib combined with toripalimab].

The results (**Figure 4A**) of monitoring gene mutations during targeted immunotherapy revealed that in addition to the original mutations, more gene mutations were found in tissues after treatment, indicating an increase in subcloning. Whether these genes play a role in drug resistance remains to be further explored. The overall change in gene mutation abundance before and after targeted immunotherapy showed a stable or declining trend (**Figure 4B**), and wilcoxon test was uesd to compare the gene abundance levels in the patient’s samples before and after treatment, and the results showed a significant difference in the median values before and after treatment. Dynamic TMB monitoring before and after treatment with solantinib combined with terriplizumab (**Figure 4C**) revealed that the PD-L1 expression was negative in patients before treatment; in contrast, TMB was highly expressed (17.5 mut/Mb in the first test and 25 mut/Mb in the second test). This study demonstrated a sustained response to immune checkpoint inhibitors.

### 3.2 Tumour microenvironment

**Figure 5:**
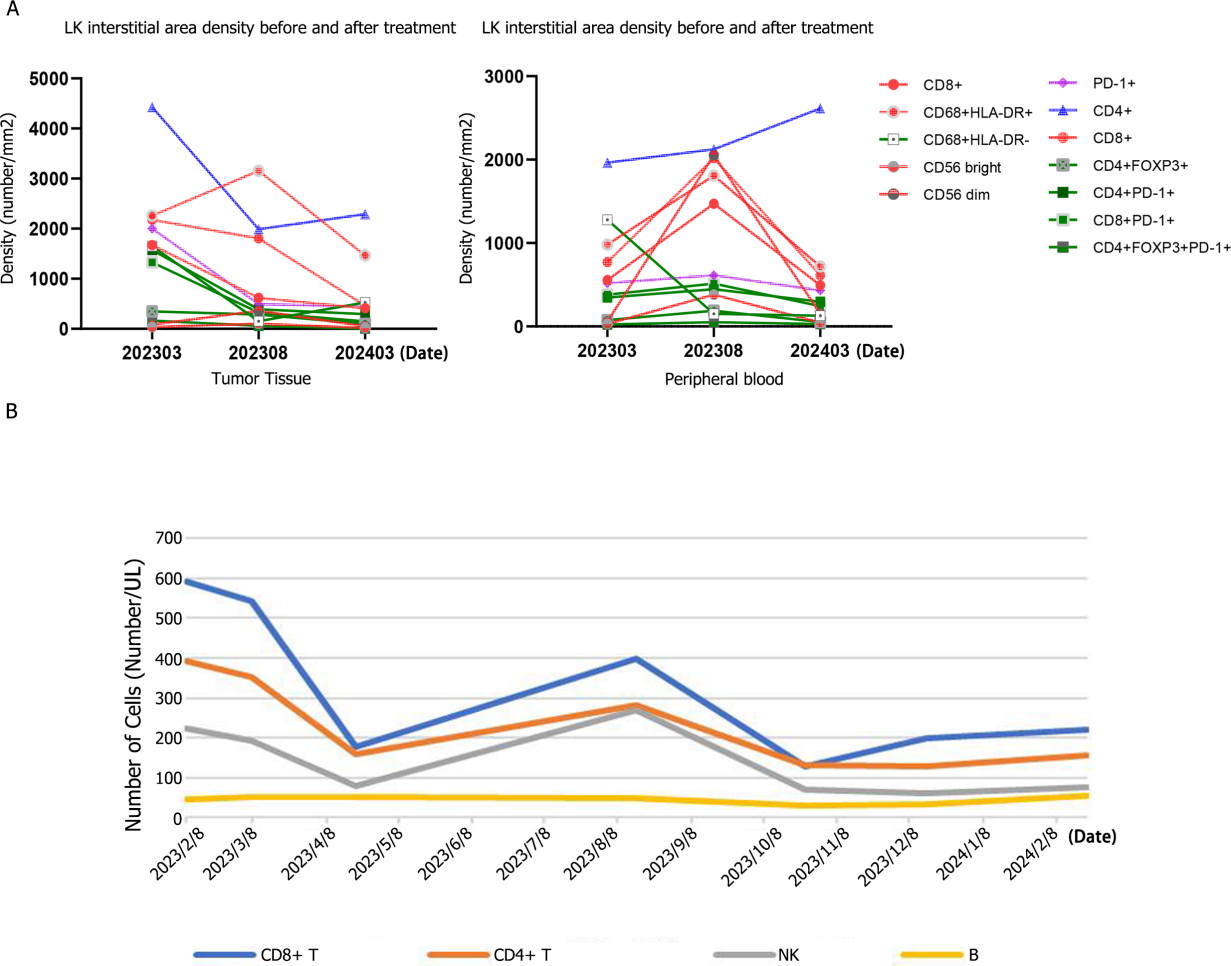
**(A)** The changes in the number of cells with cytotoxic or anti-tumor effects in the tumor parenchyma (left image) and tumor stroma (right image) before and after treatment. **(B)** Analysis of the changes in T lymphocytes, NK cells and B cells in peripheral blood before and after treatment.

The immuno-microenvironment indicators of patients were monitored during treatment (**Figure 5A**), and the results revealed that the number of cells (left, red) with cytotoxic or antitumour effects in the tumour parenchyma significantly increased and then decreased, indicating that the efficacy may have changed from strong to weak. The number of cells with depletion or regulatory effects changed slowly; that is, more cells with cytotoxic effects were observed in 2023-08, and more cells with depletion effects were observed in 2024-03, suggesting a change in efficacy. The number of cells (right, red) with cytotoxic or antitumour effects in the tumour interstitial region increased and then decreased, suggesting the possibility of a change in efficacy from strong to weak. After the depletion or regulation of cells decreased in 2023-08, that in 2024-03 continued to decrease, suggesting that these cells may be recruited to the parenchymal region to play an inhibitory role, suggesting that the efficacy was weakened. Peripheral blood analysis revealed that T lymphocytes were highly expressed in the early stage of targeted immunotherapy and then gradually decreased, suggesting that tumour-specific T cells were activated after the application of PD-1 inhibitors, thus recognizing tumour cells and attacking and killing them (**Figure 5B**).

## 4 Discussion

Pulmonary LCNEC is a relatively rare lung malignancy; however, it is commonly observed in elderly men over 65 years of age, more than 90% of whom are heavy smokers (12). The major risk factor for LCNEC is smoking, and the associated mutations are characterized by guanine-to-thymine and cytosine-to-adenine translocations. The high exon mutation profile (8.6 nonsynonymous mutations per 1 million base pairs) and high tumour mutation load (TMB) of LCNEC further support its close association with smoking (13).

LCNEC and SCLC have similar clinical and pathological features (14) The WHO has classified pulmonary LCNEC as a neuroendocrine carcinoma with a SCLC clinical phenotype (aggressive behaviour, high recurrence rate after local treatment, and similar metastasis pattern) (13). Therefore, although there are similarities between the treatment modes for pulmonary LCNEC and pulmonary SCLC, there are also differences. For resectable stage I-IIIA lung LCNEC, surgery is still the cornerstone of treatment (15), and postoperative adjuvant chemotherapy with the SCLC standard protocol (cisplatin + etoposide) can significantly improve the survival and prognosis of patients (16). However, there is currently no standard protocol for the treatment of unresectable and metastatic pulmonary LCNEC, which remains to be further explored. The patient with pulmonary LCNEC we reported was initially treated with EP chemotherapy, and the best curative effect reached PR, but the PFS was only 6 months. After the initial progression of the disease, the FOLFIRI chemotherapy regimen was used, but the efficacy was not good, and rapid progression was observed after 4 cycles of treatment. In addition, LeTreut et al. conducted a phase II prospective study in which etoposide and cisplatin were used to treat 42 patients with advanced pulmonary LCNEC, and the results revealed that the mPFS and mOS were 5.2 months and 7.7 months, respectively, after treatment with this regimen (17). Igawa et al. evaluated the efficacy of platinum-containing dual-drug chemotherapy in patients with unresectable LCNEC, and the results showed that the clinical efficacy of chemotherapy for unresectable LCNEC was comparable to that for extensive SCLC (18). Although different chemotherapy regimens can benefit patients with pulmonary LCNEC to varying degrees and delay the disease to a certain extent, overall, the survival benefits of chemotherapy alone in patients with pulmonary LCNEC are small, with a PFS of approximately 5–6 months and an OS of 7.7–12.6 months (18, 19). Therefore, it is necessary to explore new therapeutic strategies to improve the survival of patients with pulmonary LCNEC.

Neuroendocrine tumours are solid tumours with a rich blood supply, and multitarget small-molecule TKIs may be effective antitumour agents. Sovantinib has dual antiangiogenetic mechanisms and mediates the immune microenvironment, preventing tumour neovascularization by inhibiting VEGFR1/2/3 and the fibroblast growth factor receptor (FGFR1), as well as by inhibiting colony stimulating factor-1 receptor (CSF-1R), thereby reducing the number of M2-type tumour-associated macrophages (TAMs) and regulating the immune response to tumour cells (20). Two phase III clinical studies, SANET-P and SANET-EP, demonstrated good antitumour activity in advanced pancreatic neuroendocrine tumours and advanced extrapancreatic neuroendocrine tumours, with PFS times of 10.9 months and 9.2 months, respectively (21). ShenL et al (22) reported that the ORR and DCR of solantinib (250 mg) combined with toripalimab in advanced solid tumours were as high as 63.6% and 100%, respectively, and that this combination was safe and well tolerated. However, the efficacy of solantinib alone in patients with NEC G3 is still uncertain.

High TMB and PD-L1 positivity are considered predictive biomarkers of the response to ICIs. LCNEC has been reported to have a higher TMB than other NSCLC subtypes, suggesting that ICIs may be a treatment option for LCNEC. In recent years, the efficacy of ICIs in treating neuroendocrine tumours has been partially demonstrated. A multicentre retrospective study (23) included 125 patients with LCNEC, 41 of whom received ICI treatment and 84 of whom did not. The median OS of ICI patients was 12.4 months (95% CI: 10.7~23.4 months). The median OS of patients who did not receive ICIs was 6.0 months (95% CI: 4.7–9.4 months), and the difference was statistically significant (P=0.02). A single-centre retrospective analysis (24) reported the efficacy of a first-line EP regimen combined with atezolizumab in 9 LCNEC patients, and the results revealed that the ORR was 75%. The mPFS was 6.85 months, and the median duration of response (DoR) was 5.5 months. LCNEC immunotherapy combined with chemotherapy is beneficial; however, large prospective clinical studies are lacking.

Whether chemotherapy, targeting or immunization is used, the therapeutic effect on LCNEC is not completely satisfactory, and a combination of chemoimmunotherapy and targeted immunotherapy is needed to improve the efficacy and survival of patients. The results of a variety of tumour studies have suggested that the combination of targeted and anti-PD-1/PD-L1 drugs may have potential synergistic antitumour activity (25–28). In the present case, the patient achieved a PR after treatment with solantinib in combination with toripalimab following two-line chemotherapy and achieved PFS for 15.1 months. After the disease progressed again, everolimus combined with toripalimab was used, and the tumour shrank. To date, the OS of the patient has reached more than 33 months. The patient tested negative for PD-L1 but had high expression of TMB (17.5 mut/Mb at the first test and 25 mut/Mb at the second test). Therefore, long PFS was achieved in the posterior-line treatment of this patient with lung LCNEC, mainly because of the discovery of TMB-H upon genetic testing, and targeted immunotherapy combination therapy was effective. These results indicate that, theoretically, immune-based combination therapy for pulmonary LCNEC patients with TMB-H can achieve individualized and precise guidance for treatment strategies. At the same time, before and after treatment, we collected data at three time points, namely, at baseline, optimal efficacy and disease progression, and dynamically monitored the immune microenvironment of the patients. Changes in cytokines, the number of immune cells, and tumour markers were dynamically monitored throughout the treatment. The results revealed that the patients’ tumour markers decreased significantly after treatment and that the tumour markers increased significantly after the disease progressed again. Cytokines and immune cells are also correlated with the curative effect. In the initial stage of treatment, the levels of proinflammatory factors (IL-6 and TNF-a) decreased significantly, and then gradually increased, which suggested that the curative effect was likely to change from strong to weak. The tumour immune microenvironment was also consistent with the therapeutic effect. At baseline, patients exhibited an immunosuppressive microenvironment (cell groups such as CD8, CD56 and CD68 with cytotoxic or antitumour effects were present at low levels), whereas the optimal therapeutic effect involved an immuno-antitumour microenvironment (CD8, CD56 and CD68 were significantly increased). When the disease progressed, the immunosuppressive microenvironment changed again (CD8, CD56, and CD68 are significantly reduced). The possible mechanism is as follows: in the early stage of treatment, antiangiogenic inhibitors cause T cells to accumulate around tumour tissues, and at this time, tumour cells express high levels of PD-L1, resulting in an immunosuppressive state. The combination of PD-1 inhibitors can activate immune cell killing functions by increasing the immunosuppression of tumour cells. The patient in this case had TMB-H, so the curative effect was good, and a long PFS was obtained, indicating that targeted immunotherapy can have a synergistic effect on lung LCNEC. However, whether it is present only in a specific LCNEC population (TMB-H), or has a population effect remains to be verified in more patients.

The relationships among tumour antiangiogenic effects, ICI immunotherapy and changes in the internal environment of tumours are complex. More systematic evaluation methods are needed to accurately determine the benefit of targeted immunotherapy in patients with lung LCNEC. The use of reliable biomarkers to select the advantageous population for combination therapy, the selection of antiangiogenic drugs and immunotherapy drugs, the determination of the cycle and sequence of administration, the adjustment of drug doses, and the safety of combination therapy need to be explored in the future.

## 5 Conclusion

In summary, we have reported the case of a patient with advanced pulmonary LCNEC with a high TMB, whose disease progressed after front-line chemotherapy, and the treatment efficacy reached PR, with a PFS of 15.1 months after treatment with solantinib combined with toripalimab. During treatment, the cell population in the tumour parenchymal area with cytotoxic or antitumour effects increased significantly and then decreased, indicating the possibility that the efficacy ranged from strong to weak. This report highlights the efficacy of targeted immunotherapy in patients with high-TMB metastatic LCNEC and suggests that immuno-microenvironment analysis may facilitate the personalization of ICI treatment.

In the future, we aim to collaborate with additional medical institutions to recruit a larger cohort of LCNEC patients for randomized, controlled phase III clinical trials. Our goal is to identify the optimal combination regimens and discover biomarkers that can predict treatment efficacy, thereby enabling better identification of patients who would benefit most.

## Data Availability

The original contributions presented in the study are included in the article/Supplementary Material. Further inquiries can be directed to the corresponding author.
