# Correction: Case Report: Curative effect and mechanism of surufatinib combined with toripalimab in the treatment of 1 case of lung large-cell neuroendocrine carcinoma

**DOI:** 10.3389/fonc.2025.1730518

**Published:** 2025-11-18

**Authors:** Shuanglong Xiong, Chengxiang Yang, Kaiwen Fu, Xingyun Liao, Yao Ding, Kexue Zhou, Li Zhang, Yan Teng, Chunman Wu, Yongsheng Li, Houjie Liang, Yan Li

**Affiliations:** 1 Department of Oncology, Chongqing University Cancer Hospital, Chongqing, China; 2 Nanjing Geneseeq Technology Inc, Nanjing, China; 3 Department of Oncology and Southwest Cancer Centre, Southwest Hospital, Army Medical University (Third Military Medical University), Chongqing, China

**Keywords:** lung large-cell neuroendocrine carcinoma, Surufatinib, toripalimab, immune microenvironment, TMB

Author “Chunman Wu” was erroneously assigned to affiliation “Chongqing University Cancer Hospital”. This affiliation has now been removed for author “Chunman Wu”.

Affiliation “Nanjing Geneseeq Technology Inc, Nanjing, China” was omitted for author “Chunman Wu”. This affiliation has now been added for author “Chunman Wu”. Shuanglong Xiong^1†^, Chengxiang Yang^1†^, Kaiwen Fu^1^, Xingyun Liao^1^, Yao Ding^1^, Kexue Zhou^1^, Li Zhang^1^, Yan Teng^1^, Chunman Wu^2^, Yongsheng Li^1*^, Houjie Liang^3*^ and Yan Li^1*^

^1^Department of Oncology, Chongqing University Cancer Hospital, Chongqing, China, ^2^Nanjing Geneseeq Technology Inc, Nanjing, China, ^3^Department of Oncology and Southwest Cancer Centre, Southwest Hospital, Army Medical University (Third Military Medical University), Chongqing, China.

The **Conflict of interest** statement has been correct to *“*Author CW was employed by Nanjing Geneseeq Technology Inc. The remaining authors declare that the research was conducted in the absence of any commercial or financial relationships that could be constructed as a potential conflict of interest.”

The funder “1. Beijing Xisike Clinical Oncology Research Foundation (Y-HH202102-0200); 2. Senior Medical Talents Program of Chongqing for Young and Middle-aged (2020GDRC005)” were erroneously omitted.

The original version of this article has been updated.

